# miR-375 is involved in Hippo pathway by targeting YAP1/TEAD4-CTGF axis in gastric carcinogenesis

**DOI:** 10.1038/s41419-017-0134-0

**Published:** 2018-01-24

**Authors:** Wei Kang, Tingting Huang, Yuhang Zhou, Jinglin Zhang, Raymond W. M. Lung, Joanna H. M. Tong, Anthony W. H. Chan, Bin Zhang, Chi Chun Wong, Feng Wu, Yujuan Dong, Shiyan Wang, Weiqin Yang, Yi Pan, Wing Po Chak, Alvin H. K. Cheung, Jesse C. S. Pang, Jun Yu, Alfred S. L. Cheng, Ka Fai To

**Affiliations:** 1 Department of Anatomical and Cellular Pathology, State Key Laboratory of Oncology in South China, Prince of Wales Hospital, The Chinese University of Hong Kong, Hong Kong, SAR PR China; 2 Institute of Digestive Disease, Partner State Key Laboratory of Digestive Disease, The Chinese University of Hong Kong, Hong Kong, SAR PR China; 3 Li Ka Shing Institute of Health Science, Sir Y.K. Pao Cancer Center, The Chinese University of Hong Kong, Hong Kong, SAR PR China; 4 Shenzhen Research Institute, The Chinese University of Hong Kong, Shenzhen, PR China; 5 Department of Gastroenterology, The Affiliated Drum Tower Hospital of Nanjing University, Medical School, Nanjing, PR China; 6 School of Biomedical Sciences, The Chinese University of Hong Kong, Hong Kong, SAR PR China; 7 Department of Medicine and Therapeutics, The Chinese University of Hong Kong, Hong Kong, SAR PR China

## Abstract

miR-375 is a tumor-suppressive microRNA (miRNA) in gastric cancer (GC). However, its molecular mechanism remains unclear. The aim of this study is to comprehensively investigate how miR-375 is involved in Hippo pathway by targeting multiple oncogenes. miR-375 expression in gastric cancer cell lines and primary GC was investigated by qRT-PCR. The regulation of YAP1, TEAD4, and CTGF expression by miR-375 was evaluated by qRT-PCR, western blot, and luciferase reporter assays, respectively. The functional roles of the related genes were examined by siRNA-mediated knockdown or ectopic expression assays. The clinical significance and expression correlation analysis of miR-375, YAP1, and CTGF were performed in primary GCs. TCGA cohort was also used to analyze the expression correlation of YAP1, TEAD4, CTGF, and miR-375 in primary GCs. miR-375 was down-regulated in GC due to promoter methylation and histone deacetylation. miR-375 downregulation was associated with unfavorable outcome and lymph node metastasis. Ectopic expression of miR-375 inhibited tumor growth in vitro and in vivo. Three components of Hippo pathway, YAP1, TEAD4 and CTGF, were revealed to be direct targets of miR-375. The expression of three genes showed a negative correlation with miR-375 expression and YAP1 re-expression partly abolished the tumor-suppressive effect of miR-375. Furthermore, CTGF was confirmed to be the key downstream of Hippo-YAP1 cascade and its knockdown phenocopied siYAP1 or miR-375 overexpression. YAP1 nuclear accumulation was positively correlated with CTGF cytoplasmic expression in primary GC tissues. Verteporfin exerted an anti-oncogenic effect in GC cell lines by quenching CTGF expression through YAP1 degradation. In short, miR-375 was involved in the Hippo pathway by targeting YAP1-TEAD4-CTGF axis and enriched our knowledge on the miRNA dysregulation in gastric tumorigenesis.

## Background

Gastric cancer (GC) is a heterogeneous disease and its mechanisms of development remain poorly understood. It is one of the common malignancies and the second most frequent cause of cancer-related death worldwide with a high incidence rate in Eastern Asian countries. Many risk factors are strongly correlated with gastric carcinogenesis, including *Helicobacter pylori* or EBV infection, high-salt and low-vegetable diet, smoking, intestinal metaplasia, and the host genetic susceptibility SNPs^1^. GC is traditionally classified into two histological types: intestinal type and diffuse type according to the morphological changes of the cancer cells. To better reveal the molecular mechanism of GC, the Cancer Genome Atlas (TCGA) provided a new molecular classification of GC consisting of four molecular subtypes: EBV-positive GC, microsatellite unstable tumors (MSI), genomically stable tumors (GS), and tumors with chromosomal instability (CIN)^2^. Each subtype has its distinct molecular features which strongly correlate with a different origin of gastric carcinogenesis.

In gastric tumorigenesis, multiple signaling pathways are deregulated due to genetic or epigenetic alterations. Hippo, a signaling pathway that controls cell growth and organ size, has been confirmed to be correlated with tumor initiation^3,4^. As the key downstream mediator of Hippo pathway, YAP1 is activated in multiple cancer types and functions as a driver oncogene, even bypassing oncogenic RAS signaling^5–8^. YAP1 functions as a transcription co-activator and TEAD transcription factors are the main binding partner for YAP1, together they exert oncogenic roles in tumorigenesis. However, the downstream players of YAP1/TEAD complex in GC are unidentified. Emerging evidence also indicates that the Hippo-YAP1 pathway is under control by the deregulated microRNA (miRNA) network^9,10^.

miRNAs play an important role in tumor initiation and progression. To comprehensively elucidate the deregulated expression of miRNAs in GC, we performed miRNA expression profiling using GC cell lines and miR-375 was found to be among the top-10 down-regulated miRNAs^9,11^. Although miR-375 has been reported to play a tumor-suppressive role in GC by targeting JAK2^12,13^, ERBB2^14^, PDK1, and 14-3-3zeta^15^, more important targets need to be identified in order to comprehensively reveal the key role of miR-375 in gastric carcinogenesis. In this study, we reported for the first time that in GC miR-375 functions as a master controller of Hippo-YAP1 signaling by targeting multiple oncoproteins.

## Results

### miR-375 is down-regulated and exerts tumor suppressor function in GC

**Fig. 1:**
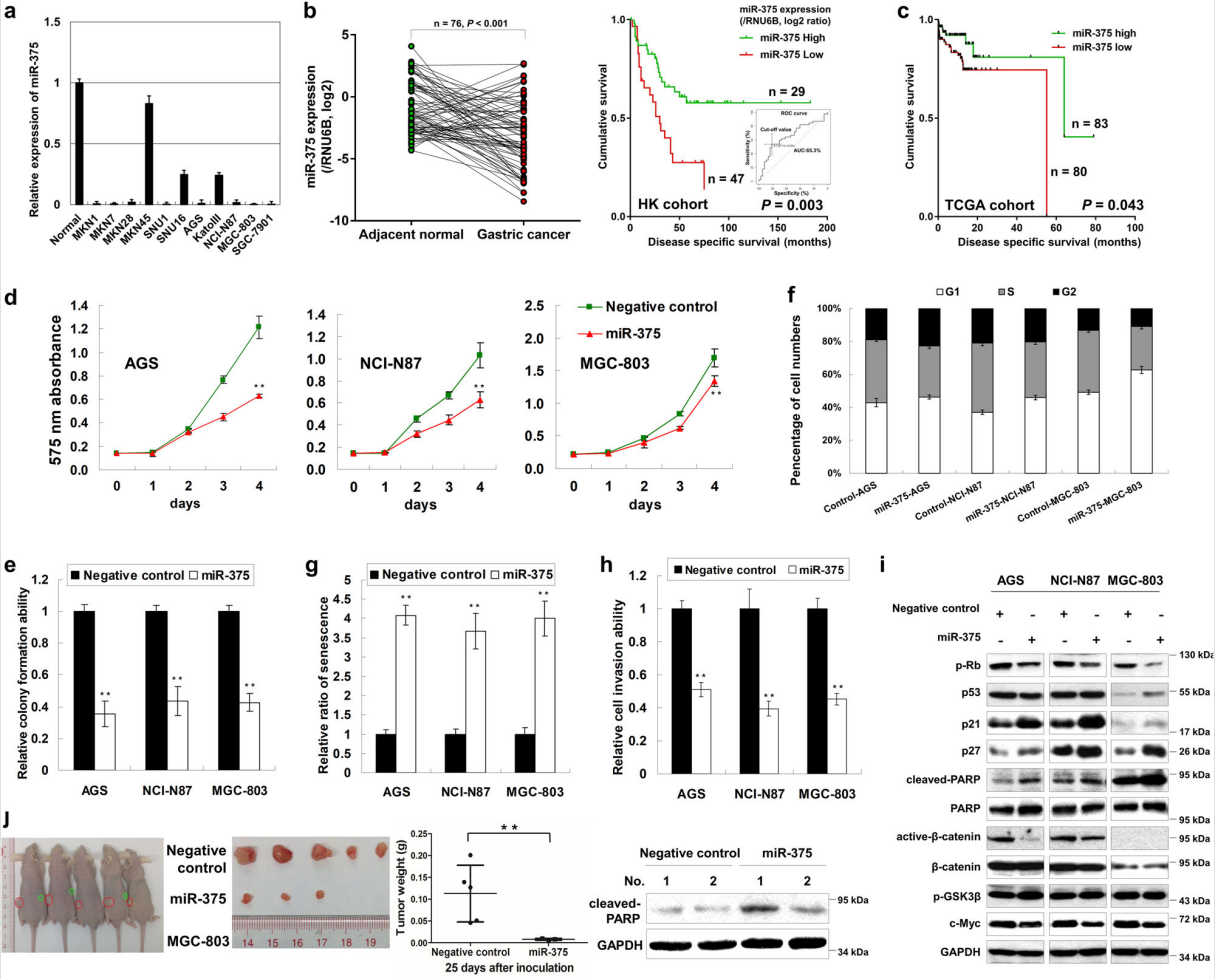
miR-375 is down-regulated and exerts a tumor suppressor function in GC **a** miR-375 showed decreased expression in eleven GC cell lines compared with normal gastric epithelium tissue. **b** miR-375 is down-regulated in tumor tissues compared with adjacent non-tumorous tissues (*n* = 76, *P* *<* 0.001) (left panel). Low miR-375 expression (defined by ROC curve) correlates with poor disease specific survival in GC (*P* *=* 0.003) (right panel). **c** Low miR-375 expression was associated with a poor disease free survival in TCGA cohort (*P* = 0.043). **d** 4-day MTT proliferation results after ectopic expression of miR-375 in AGS, NCI-N87, and MGC-803 cells (**, *P* < 0.001). The error bars represented the standard deviations (SDs). **e** Ectopic expression of miR-375 inhibited monolayer colony formation in AGS, NCI-N87, and MGC-803 cells (**, *P* *<* 0.001). The experiment was performed independently three times for standard deviations (SDs). **f** Flow cytometry analysis of miR-375 transfectants compared with scramble miRNA transfectants. **g** Ectopic expression of miR-375 induced senescence in a 3-day transfection assay (**, *P* *<* 0.001). SDs were achieved by the normalization of positive cell number in 3 random vision fields under a microscope. **h** The invasive ability was significantly impaired in miR-375 treated cells compared with scramble miRNA counterparts (**, *P* *<* 0.001). **i** miR-375 overexpression induced G1-phase cell cycle arrest, late apoptosis and suppression of Wnt/β-catenin signaling pathway, which was indicated by Western blot analysis of p21, p27, p-Rb, cleaved-PAPR, active-β-catenin, and c-Myc. **j** Stable-expression of miR-375 inhibited xenograft formation using MGC-803 cell model (left and middle panel, *n* = 5, **, *P* *<* 0.001) and induced late apoptosis in xenografts (right panel)

miR-375 expression was uniformly down-regulated in 11 GC cell lines compared with the normal gastric epithelium (Fig. 1a). Then, 4 GC cell lines, AGS, NCI-N87, MGC-803, and MKN1, were treated with 5-Aza, TSA, or 5-Aza/TSA. miR-375 expression was significantly restored in the drug treatment groups especially in the 5-Aza/TSA combination group, suggesting promoter methylation and histone deacetylation are co-responsible for miR-375 downregulation in GC (Supplementary Figure S1a). In a total of 76 paired primary RNA samples, miR-375 was found to be down-regulated in 57 (75.0%) tumor tissues when compared to adjacent normal gastric tissues (*P* *<* 0.001, left panel of Fig. 1b). Then two groups were stratified according to receiver operator characteristic (ROC) curve. Patients with low miR-375 expression (*n* = 49) showed a poorer survival compared with those with high expression (*n* = 27, *P* *=* 0.029, right panel of Fig. 1b). This result was concordant with TCGA data (http://cancergenome.nih.gov/): in this large cohort, miR-375 low-expression also correlates with a shorter survival in GC patients (*P* *=* 0.043, Fig. 1c).

Clinicopathologic correlation of miR-375 was further analyzed. Supplementary Table S1 summarized the correlation of miR-375 with other clinicopathologic parameters in GC patients. The decreased expression of miR-375 was correlated with advanced stage (*P* *=* 0.002) and lymph node metastasis (*P* *=* 0.001), suggesting that miR-375 downregulation might be involved in GC metastasis (Supplementary Table S1). By univariate Cox regression analysis, sex, histological type, stage, lymph node metastasis and miR-375 were correlated with prognosis, but by multivariate analysis, only advanced stage was associated with poor prognosis independently (*P* *=* 0.001, Supplementary Table S2).

As miR-375 is often down-regulated in GC, we next investigated its functional role in GC cell lines. Ectopic miR-375 expression suppressed cell proliferation of AGS, NCI-N87, and MGC-803 GC cells in a 4-day MTT assay (*P* *<* 0.001, Fig. 1d). The tumor suppressive effect of miR-375 was further validated by monolayer colony formation with a significant reduction of colony numbers in miR-375 transfectants compared with scramble miRNA groups (*P* *<* 0.001, Fig. 1e and Supplementary Figure S1b). As miR-375 inhibited cell growth, we next evaluated the possible underlying mechanisms. Ectopic expression of miR-375 resulted in G1 cell cycle arrest 24 h after transfection (AGS, from 40.2 to 46.2%; NCI-N87, from 36.1 to 46.2%; MGC-803, from 47.6 to 62.1%). The percentage of S-phase cells decreased accordingly in miR-375 transfectants (Fig. 1f and Supplementary Figure S1c). Ectopic expression of miR-375 also induced senescence in a 3-day transfection assay (*P* *<* 0.001, Fig. 1g and Supplementary Figure S1d), which was concordant with G1-phase cell cycle arrest. In addition, ectopic expression of miR-375 significantly suppressed the invasive abilities of GC cells (*P* *<* 0.001, Fig. 1h and Supplementary Figure S1e). Western blot analysis revealed the decreased phosphorylation of retinoblastoma protein (p-Rb) and elevated p21 and p27 in miR-375 ectopic expression transfectants, reflecting G1-phase cell cycle arrest. The cleaved-PARP showed activated form, indicating that miR-375 induced late apoptosis in GC cells. In addition, the Wnt/β-catenin signaling pathway was suppressed by miR-375 which was indicated by decreased expression of active-β-catenin and c-Myc (Fig. 1i). To further explore the role of miR-375 on tumor growth in vivo, MGC-803 cells, which could form tumors in nude mice, was employed for the animal model study. MGC-803 cells with or without stable-expression miR-375 were inoculated into the dorsal flank of nude mice. 25 days later, the size of xenografts with ectopic expression of miR-375 was significantly smaller than the control group (*P* *<* 0.001). Moreover, elevated cleaved-PARP was detected in the xenograft samples of miR-375 overexpression (Fig. 1j).

Finally, we checked the correlation of miR-375 expression with GC molecular classification in TCGA cohort. miR-375 downregulation was strongly associated with an EBV-positive subtype, which displayed whole-genomic DNA hypermethylation compared with the other three molecular subtypes (*P* *<* 0.0001, Supplementary Figure S1f)^2^.

### miR-375 targets YAP1, TEAD4, and CTGF in GC

**Fig. 2:**
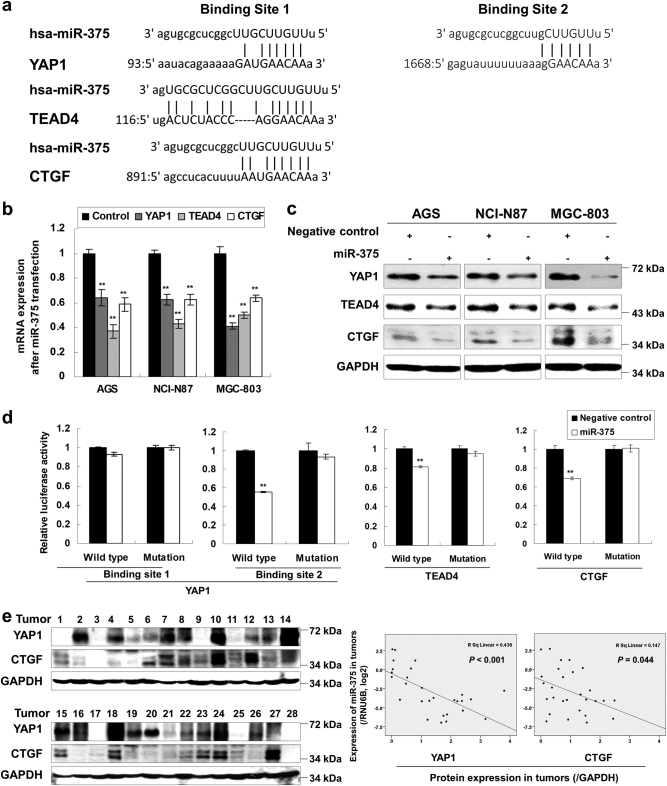
miR-375 directly targets YAP1, TEAD4, and CTGF in GC **a** The putative miR-375 binding sites in the 3’UTR of YAP1, TEAD4, and CTGF as predicted by www.microrna.org and www.targetscan.org. **b** mRNA expression of YAP1, TEAD4, and CTGF after miR-375 transfection in AGS, NCI-N87, and MGC-803 cells (**, *P* *<* 0.001). **c** miR-375 ectopic expression decreased YAP1, TEAD4, and CTGF protein expression in AGS, NCI-N87, and MGC-803 cells. **d** miR-375 suppressed the relative luciferase activity in the constructs which contain the wild type sequence of the binding site in the 3’UTR of YAP1 (only binding site 2), TEAD4, and CTGF (*, *P* *<* 0.05; **, *P* *<* 0.001). Wild type, luciferase construct containing wild type binding site in YAP1 3’UTR; Mutation, mutated nucleotides were introduced to the complementary seed sequence. **e** YAP1 and CTGF protein expression showed negative correlation with miR-375 expression in primary gastric tumors (*n* = 28, *P* *<* 0.001 and *P* *=* 0.044, respectively)

By bioinformatics analysis (www.microrna.org and www.targetscan.org), miR-375 was found to have several putative targets including YAP1 (two binding sites for miR-375 in its 3’UTR), TEAD4, and CTGF which belong to key oncogenic downstream mediators of Hippo signaling pathway (Fig. 2a). To investigate the possible regulatory effect of miR-375 on YAP1/TEAD4/CTGF, both mRNA and protein expression were examined. Both mRNA and protein expression were found to be down-regulated in AGS, NCI-N87, and MGC-803 cells following miR-375 ectopic expression (*P* *<* 0.001, Fig. 2b and c). The results were also confirmed in MKN28 and SGC-7901 cells (*P* *<* 0.001, Supplementary Figures S2a and b).

To test whether these three proteins are the direct targets of miR-375, a series of luciferase assays were performed. The fragments of the YAP1 3’UTR containing the predicted or mutant miR-375 binding site 1 and 2 were subcloned into the pMIR-REPORT vector. We found that miR-375 exerted a significant inhibitory effect on the luciferase activity in the construct which contains the wild type sequence of binding site 2, whereas no suppressive effects were observed in binding site 1 and mutation constructs. Similarly, miR-375 expression significantly decreased the luciferase activity of the constructs with the wild type binding sequences in 3’UTR of TEAD4 and CTGF (*P* *<* 0.001, Fig. 2d). These results revealed that miR-375 specifically and directly suppressed YAP1, TEAD4, and CTGF expression by binding with their 3’UTRs.

We then examined YAP1 or CTGF protein expression correlation with miR-375 in 28 frozen primary tumor samples. YAP1 and CTGF protein expression were significantly negatively correlated with miR-375 expression in clinical samples (*P* *<* 0.001 and *P* *=* 0.044, respectively, Fig. 2e), suggesting YAP1 and CTGF were up-regulated in GC development, at least in part, due to the silence of miR-375 expression.

### Inhibition of miR-375 exerts oncogenic role in GC

**Fig. 3:**
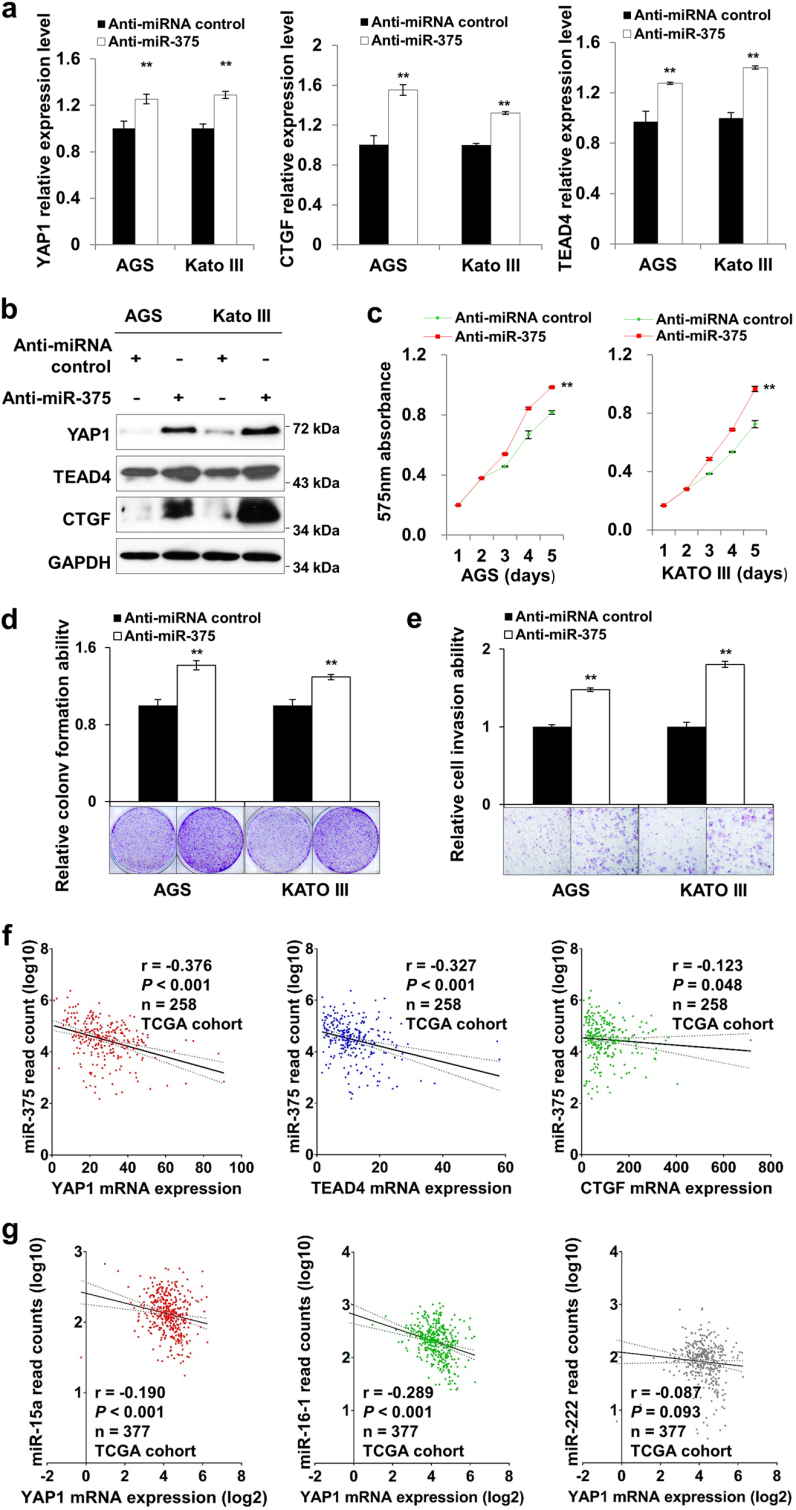
miR-375 knockdown exerts oncogenic property in GC cell lines **a** mRNA expression of YAP1, TEAD4, and CTGF after miR-375 knockdown in AGS and Kato III cells. **b** Anti-miR-375 up-regulated YAP1, TEAD4, and CTGF protein expression. **c** Anti-miR-375 promoted cell proliferation in a 5-day MTT proliferation assays (**, *P* < 0.001). **d** Monolayer colony formation ability was significantly enhanced by Anti-miR-375 in GC cells (**, *P* < 0.001). **e** The cell invasive ability was promoted by Anti-miR-375 compared with Anti-miRNA control counterparts (**, *P* < 0.001). **f** Expression correlation of miR-375 with YAP1, TEAD4, and CTGF mRNA expression in TCGA cohort (*n* = 258). YAP1 (*r* = −0.376, *P* < 0.001), TEAD4 (*r* = -0.327, *P* < 0.001), CTGF (*r* = −0.123, *P* = 0.048) showed negative correlation with miR-375 in primary GC samples. **g** Expression correlation of YAP1 mRNA expression with miR-15a (*r* = −0.190, *P* < 0.001), miR-16-1 (*r* = −0.289, *P* < 0.001) and miR-222 (*r* = −0.087, *P* *=* 0.093) in TCGA cohort

The tumor-suppressive role of miR-375 was further validated by knockdown assays in AGS and Kato III cells. miR-375 knockdown by Anti-miR-375 up-regulated mRNA expression of YAP1, TEAD4, and CTGF in some GC cell lines (Fig. 3a). Uniformly, protein expression of these three targets were up-regulated after Anti-miR-375 transfection (Fig. 3b). Functional studies were performed after ectopic Anti-miR-375 expression. Anti-miR-375 treatment increased cell proliferation rate in a 5-day MTT proliferation assay (Fig. 3c). miR-375 knockdown also led to the enhancement of colony formation ability in GC cell lines (*P* *<* 0.001, Fig. 3d). Consistently, the cell invasion ability was significantly enhanced after Anti-miR-375 transfection (*P* *<* 0.001, Fig. 3e). To confirm the inhibitory effect of miR-375 on YAP1, TEAD4, and CTGF in primary GC samples, the expression correlation in TCGA cohort were analyzed. As shown in Fig 3f, miR-375 showed negative correlation with YAP1 (*r* = −0.376, *P* < 0.001), TEAD4 (*r* = −0.327, *P* *<* 0.001), and CTGF (*r* = −0.123, *P* = 0.048) respectively. As miR-15a, miR-16 and miR-222 have been reported to be associated with YAP1 expression^9,16^, we also analyzed the expression correlation of these 3 miRNAs with YAP1 to compare with the weight of miR-375. Although miR-15a (*r* = −0.190, *P* *<* 0.001) and miR-16-1 (*r* = −0.289, *P* *<* 0.001) showed negative correlation with YAP1 (Fig 3g), miR-375 was still the most effective regulator for YAP1 with a more stringent Pearson correlation coefficient “*r* = −0.376”.

### YAP1 re-expression partly abrogates the tumor suppressive effect of miR-375 in GC

**Fig. 4:**
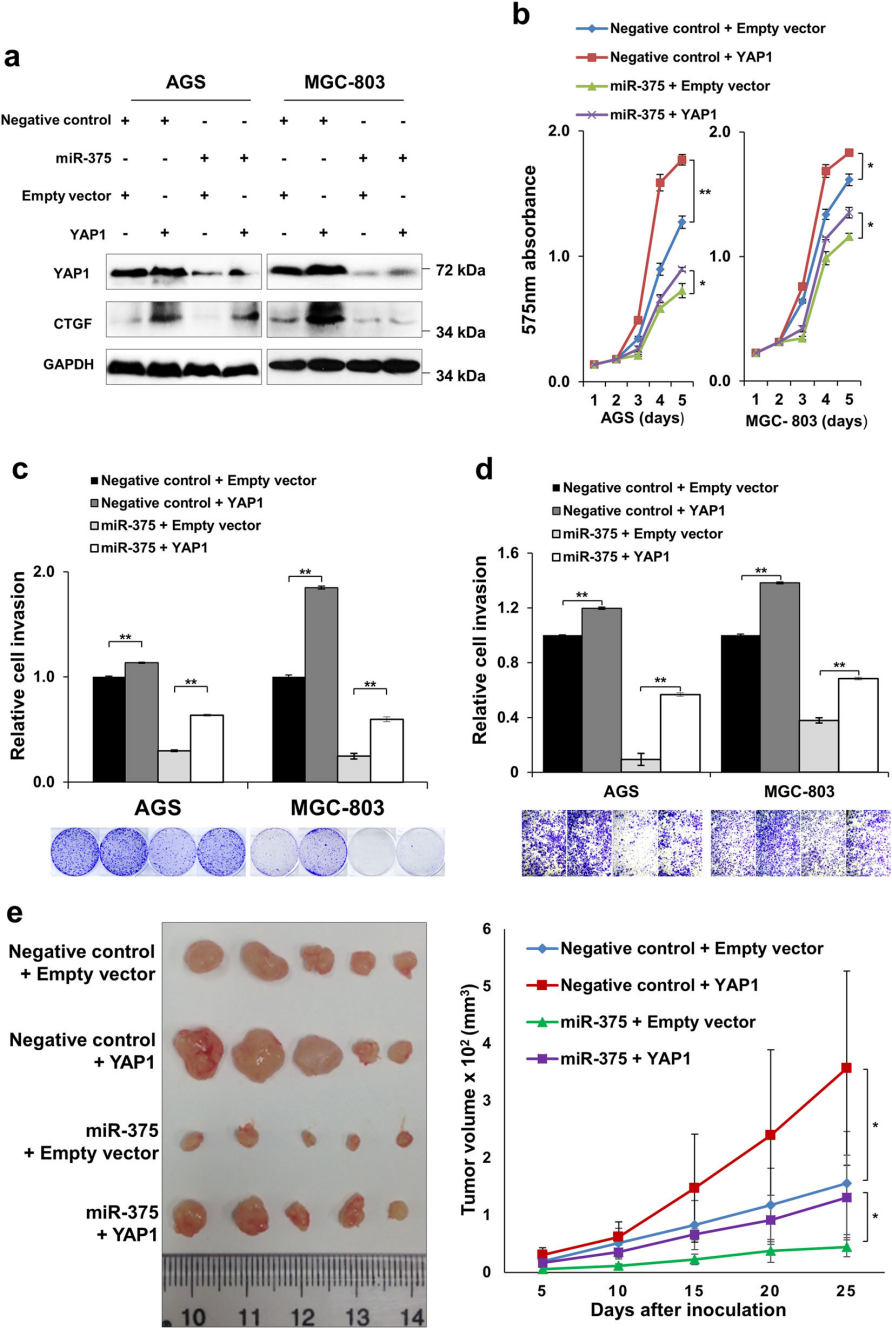
YAP1 re-expression partly abrogates the tumor suppressive effect of miR-375 in GC cells **a** Western blot analysis of YAP1 and CTGF in the rescue experiments. **b** YAP1 overexpression promoted cell proliferation compared with Empty vector control. Concordantly, YAP1 re-expression in AGS and MGC-803 cells enhanced proliferation in the presence of miR-375 (*, *P* *<* 0.05; **, *P* *<* 0.001). **c** Monolayer colony formation assays revealed that YAP1 overexpression or re-expression promoted colony formation compared with Empty vector control or miR-375 alone group respectively (**, *P* *<* 0.001). **d** The impaired cell invasive ability was partly restored in YAP1 re-expression group compared with a miR-375 alone group (**, *P* *<* 0.001). **e** In xenograft formation assay by MGC-803 cells, YAP1 re-expression promoted xenograft formation compared with miR-375 alone group (*, *P* *<* 0.05)

As YAP1 has been confirmed to be a direct target of miR-375, we further investigated if YAP1 re-expression rescued the inhibitory phenotypic changes caused by miR-375. YAP1 was re-overexpressed in AGS and MGC-803 cells after treatment with miR-375 (Fig. 4a). The growth suppressive effect of miR-375 was partially abrogated by YAP1 re-expression (MTT proliferation assay, Fig. 4b; monolayer colony formation assays, Fig. 4c), indicating that YAP1 was involved in miR-375-induced suppression of cell growth. Meanwhile, YAP1 re-expression significantly enhanced cell invasion compared with miR-375 alone group (*P* *<* 0.001, Fig. 4d). Notably, tumorigenicity assay in animal model revealed that MGC-803 cells co-transfected with miR-375 and YAP1 formed larger xenografts than those with miR-375 alone (*P* *<* 0.05, Fig. 4e).

### CTGF is regulated by YAP1 and TEADs in GC

**Fig. 5:**
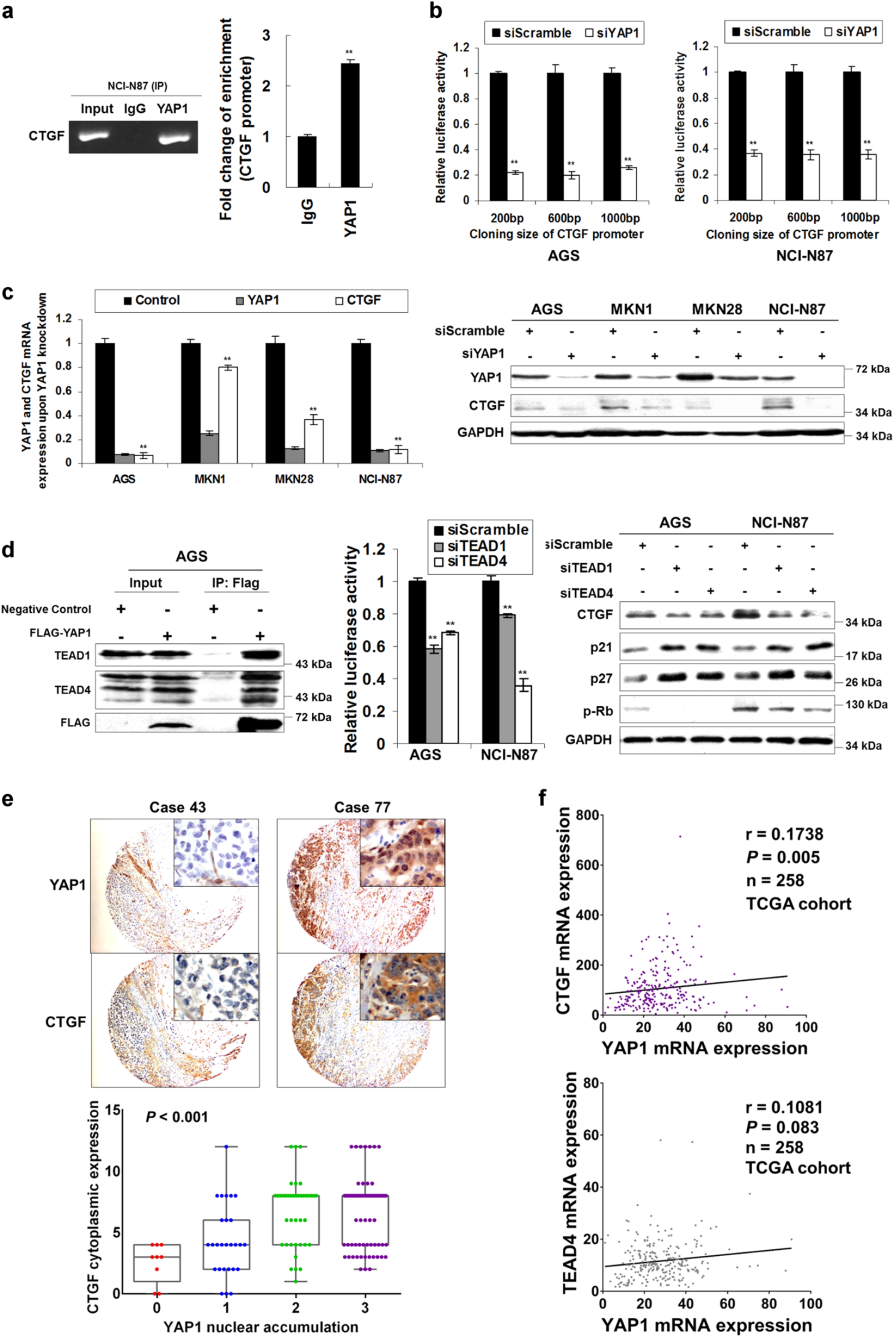
CTGF is directly regulated by YAP1 and TEADs in GC **a** ChIP-PCR and ChIP-qPCR revealed CTGF is a direct target of YAP1. YAP1-IP showed 2.43 times enrichment compared with non-specific IgG-IP for CTGF promoter binding affinity (**, *P* *<* 0.001). **b** Luciferase activities were suppressed by siYAP1 in the constructs which contained CTGF promoter sequences with different fragment size using AGS and NCI-N87 cells (**, *P* *<* 0.001). **c** qRT-PCR and Western blot of CTGF after YAP1 knockdown in AGS, MKN1, MKN28, and NCI-N87 cells (**, *P* *<* 0.001). **d** Immunoprecipitation of FLAG-YAP1 and TEAD1/4 detection in AGS cells (left panel). siTEAD1 or siTEAD4 decreased the luciferase activity after transfection with constructs containing CTGF promoter region in AGS and NCI-N87 cells (middle panel, **, *P* *<* 0.001). The Western blot analysis of CTGF, p21/p27, and p-Rb after TEAD1 and TEAD4 knockdown (right panel). **e** Representative IHC images of CTGF and YAP1 expression in the same GC sample (Case 43, both YAP1 and CTGF show weak expression. Case 77, YAP1 strongly accumulates in the nuclei and CTGF exhibits strong cytoplasmic staining.). Moderate/strong CTGF cytoplasmic staining showed a positive correlation with YAP1 nuclear accumulation (*P* *<* 0.001). **f** YAP1 and CTGF mRNA expression showed a positive correlation (upper panel, *P* = 0.005) but YAP1 mRNA expression was not significantly associated with TEAD4 expression (lower panel, *P* *=* 0.083) in TCGA cohort

ChIP-PCR and ChIP-qPCR were employed to investigate CTGF as the direct downstream target of YAP1. YAP1-IP showed 2.43 times enrichment compared with non-specific IgG-IP for CTGF promoter binding affinity (*P* *<* 0.001, Fig. 5a). Based on bioinformatics analysis, 3 TEAD-bound motifs (GGAATG) were predicted within 200 bp from the transcription start site of CTGF (Supplementary Figure S3a). Three fragments of CTGF promoter with different sizes (200, 600, 1000 bp upstream of the transcription starting site of CTGF) were subcloned into the pGL3-Basic vector. siYAP1 significantly suppressed luciferase activity in all the constructs (*P* *<* 0.001, Fig. 5b). To further investigate the regulation of CTGF expression by YAP1, 4 GC cell lines were transfected with siYAP1, and CTGF expression was examined. CTGF showed decreased mRNA and protein expression upon YAP1 knockdown (*P* *<* 0.001, Fig. 5c). As TEAD transcription factor family are the main binding partner for YAP1, we then assessed if TEADs also regulate CTGF expression. Co-IP assay confirmed the direct interaction of YAP1 with TEADs in GC (left panel of Fig. 5d). Luciferase assays revealed TEAD1 and TEAD4 regulate CTGF expression by direct binding with its promoter (*P* *<* 0.001, middle panel of Fig. 5d). siTEAD1/4 down-regulated CTGF expression and induced G1-phase cell cycle arrest which was indicated by Rb hypo-phosphorylation and p21/p27 activation (right panel of Fig. 5d). Functional studies demonstrated siYAP1, siTEAD1, and siTEAD4 suppressed GC cell proliferation in a 5-day MTT assay (*P* *<* 0.001, Supplementary Figure S3b).

Expression correlation of CTGF and YAP1 in primary GC was analyzed. As shown in Fig. 5e, CTGF often showed moderate or strong cytoplasmic staining in the cases which exhibited strong YAP1 accumulation in the nucleus. Strong CTGF cytoplasmic staining was more frequently found in YAP1 nuclear accumulation tumors (score 2+/3+) than YAP1-negative/weak tumors (score 0+/1+) (*P* *<* 0.001, Fig. 5e). Meanwhile, YAP1, CTGF, and TEAD4 expression correlation were analyzed in 258 primary tumors from TCGA^17,18^. YAP1 mRNA expression showed a positive correlation with CTGF (*P* *=* 0.005, the upper panel of Fig. 5f), but its expression was not significantly associated with TEAD4 expression (*P* *=* 0.083, lower panel of Fig. 5f).

To further confirm the regulation of CTGF by YAP1/TEAD complex, GC cell lines were treated with Verteporfin (VP), a pharmacological inhibitor of YAP1/TEAD interaction. The 3-day MTT proliferation assays validated that VP suppressed cell proliferation in a dose dependent manner in GC cell lines (Supplementary Figure S3c). YAP1, the druggable target for VP, exhibited elevated degradation after treatment. As the downstream target of YAP1, CTGF showed decreased expression as a consequence (Supplementary Figure S3d).

### CTGF knockdown exerts anti-oncogenic effect and phenocopies siYAP1 or miR-375 in GC

**Fig. 6:**
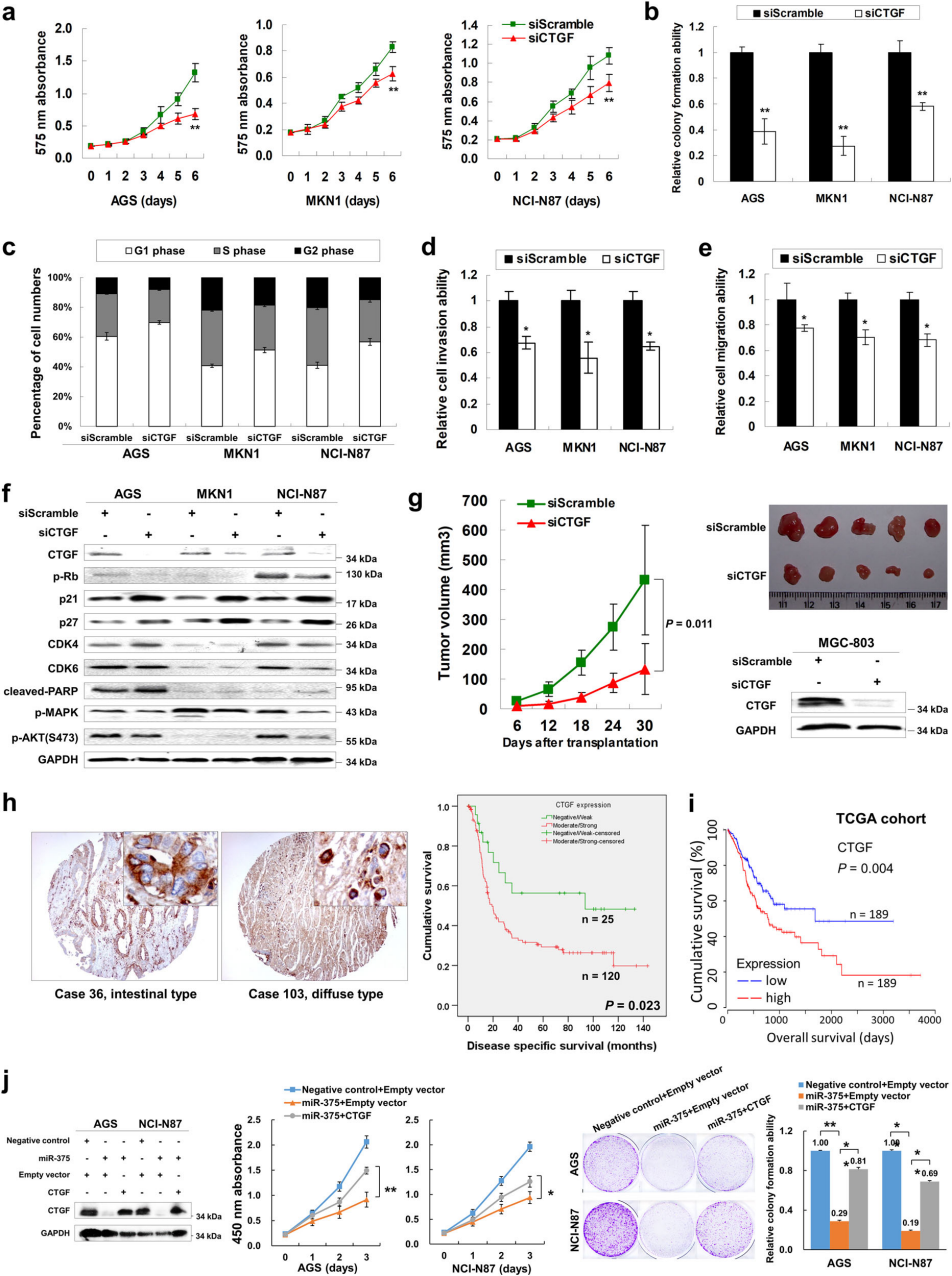
CTGF knockdown in GC cells phenocopies siYAP1 and miR-375 ectopic expression both in vitro and in vivo **a** MTT proliferation assay revealed that CTGF knockdown by siRNA significantly suppressed proliferation in GC cells (**, *P* *<* 0.001). The mean and SDs of the plots were obtained from 6 wells within 3 independent experiments. **b** Monolayer colony formation assays indicated that CTGF knockdown reduced anchorage-dependent colony formation (**, *P* *<* 0.001). The experiments were done three times and the error bars represented SDs. **c** Flow cytometry analysis revealed the accumulation of cells in G1-phase 24 h after siCTGF treatment. **d** CTGF knockdown decreased the invasive ability of the GC cells (*, *P* *<* 0.05). The cells in 3 random vision fields from 3 independent experiments were counted under the microscope and calculated for getting SDs. **e** The cell migration ability was significantly inhibited by siCTGF (*, *P* *<* 0.05). **f** Western blot analysis demonstrated p-Rb downregulation, p21/p27 upregulation, cleaved-PARP activation, MAPK and AKT signaling suppression after CTGF knockdown. **g** siCTGF-MGC-803 formed smaller xenograft tumors than siScramble-MGC-803 in a 30-day in vivo study (*P* *=* 0.011). **h** The representative CTGF IHC pictures in primary samples (Case 36, intestinal type; Case 103, diffuse type; original magnification × 100; insertion × 400). Kaplan–Meier plot of DSS according to CTGF expression indicated moderate/strong CTGF expression correlates with poor survival in gastric adenocarcinoma (right panel, *P* = 0.023). **i** High CTGF mRNA expression was associated with poor overall survival in TCGA cohort (*P* = 0.004). **j** Western blot analysis and cell functional test of CTGF in the rescue experiments (*, *P* *<* 0.05; **, *P* *<* 0.001)

Using siRNA-mediated knockdown, CTGF showed decreased expression at the mRNA (*P* *<* 0.001, Supplementary Figure S4a) and protein level in AGS, MKN1, and NCI-N87 cells. A significantly decreased proliferation was observed in the siCTGF treated group compared with scramble siRNA group in all 3 cell lines examined (*P* *<* 0.001, Fig. 6a). Monolayer colony formation assays indicated that CTGF knockdown significantly reduced colony formation ability in GC cell lines (*P* *<* 0.001, Fig. 6b and Supplementary Figure S4b). Since a growth inhibitory effect was observed in siCTGF transfected cells, we analyzed the transfectants for cell cycle parameters via flow cytometry. 24 h after transfection, accumulation of G1 cells increased in siCTGF transfectant compared with scramble siRNA controls (58.2 vs. 69.8% in AGS; 40.5 vs. 49.9% in MKN1; 41.3 vs. 55.0% in NCI-N87 cells), while S-phase cell percentage decreased after siCTGF transfection in these 3 cell lines (Fig. 6c and Supplementary Figure S4c). In addition, siRNA-mediated knockdown of CTGF decreased GC cell invasion (*P* *<* 0.05, Fig. 6d and Supplementary Figure S4d) and migration abilities (*P* *<* 0.05, Fig. 6e). The G1-phase cell cycle arrest was further confirmed by decreased p-Rb and increased p21/p27 expression. Late cell apoptosis, as determined by cleaved-PARP activation, was also validated by Western blot in the cells treated with siCTGF. CTGF knockdown also suppressed MAPK and AKT signaling activity in GC (Fig. 6f). To further investigate the effect of siCTGF on in vivo growth of the gastric tumor, siCTGF and siScramble-transfected MGC-803 cells were injected subcutaneously to the right and left dorsal flank of nude mice respectively. siCTGF transfectant formed smaller tumors on the right dorsal flank than scramble controls on the left dorsal flank 30 days after inoculation (*P* *=* 0.011, Fig. 6g).

IHC was performed to assess CTGF protein expression in 145 primary gastric adenocarcinoma samples. Expression of CTGF protein was mainly localized in the cytoplasm of tumor cells (left panel of Fig. 6h). We found moderate/strong CTGF expression (*n* = 120) was significantly correlated with shorter disease specific survival (DSS, *P* *=* 0.023, right panel of Fig. 6h). Data from TCGA cohort also indicated that high CTGF mRNA expression was associated with poorer overall survival, which kept good concordance with our findings (*P* = 0.004, Fig. 6i)^17–19^. Furthermore, after re-expression of CTGF, the inhibitory phenotype changes by miR-375 were restored in partial (Fig. 6j), which indicated that CTGF is another crucial target of miR-375.

The clinicopathologic characteristics of 145 GC patients and the association with CTGF expression were summarized in Supplementary Table S3. CTGF moderate/strong expression tumors were more likely to be found in advanced stage group (Stage III and IV, *P* *=* 0.026). Univariate analysis indicated that male sex (*P* *=* 0.004), histology with diffuse component (*P* *=* 0.002), advanced grade (*P* *=* 0.007), stage (*P* *<* 0.001), T stage (*P* *<* 0.001), N stage (*P* *<* 0.001), M stage (*P* *<* 0.001) and the presence of lymph node metastasis (*P* *<* 0.001) correlated with poor DSS. By multivariate Cox proportional hazards regression analysis, only sex (*P* *=* 0.007) and stage (*P* *<* 0.001) were independently associated with DSS (Supplementary Table S4). To elucidate the expression correlation of CTGF with the molecular classification of GC, TCGA cohort was employed for analysis^17,18^. CTGF mRNA upregulation was mostly found in GS subtype (Supplementary Figure S4e), suggesting that CTGF might play a promoting role in tumor cell metastasis.

### The summary of miRNA deregulation and their oncogenic targets in Hippo pathway

**Fig. 7:**
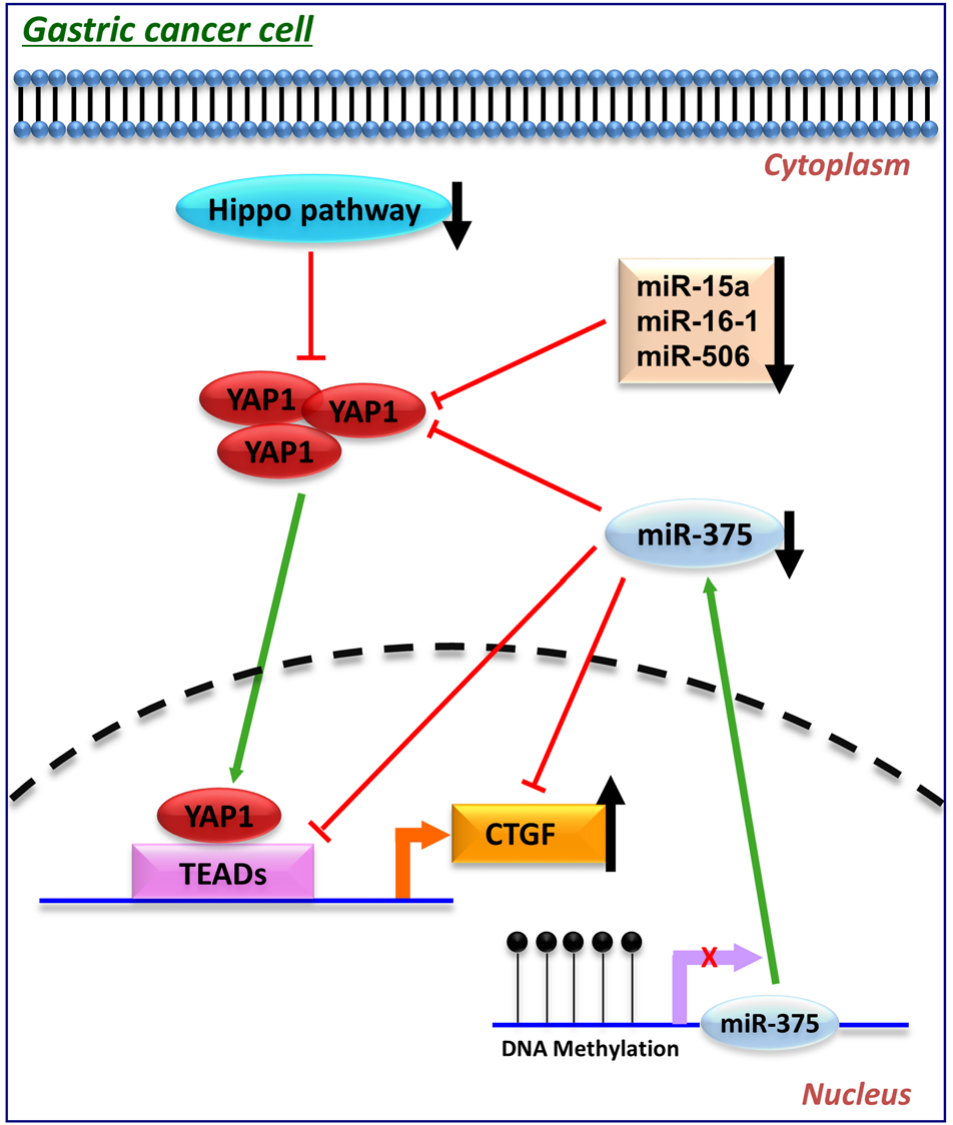
The schematic representation of the involvement of miR-375 in Hippo pathway YAP1, which is negatively regulated by miR-15a, miR-16-1 and miR-506, is up-regulated and binds with TEADs to regulate CTGF expression. miR-375, a super controller involved in Hippo pathway, directly targets YAP1, TEAD4, and CTGF and exerts a tumor suppressive function in GC

YAP1, the center of the Hippo signaling cascade, is negatively regulated by miR-15a, miR-16-1, and miR-506 in GC. Meanwhile, miR-375 targets YAP1, TEAD4, and CTGF and exerts tumor suppressor function involved in Hippo pathway. Thus, the miR-375 silence in GC activates the key downstream oncogenic components of Hippo pathway and promotes gastric tumorigenesis (Fig. 7).

## Discussion

Hippo signaling pathway is an emerging kinase cascade in gastrointestinal homeostasis and tumorigenesis^20^. As the main target for Hippo pathway, the oncogenic role of YAP1 has been extensively investigated^8,9^. In this study, we first revealed that CTGF is the key downstream effector for the oncogenic function of YAP1 in gastric carcinogenesis, and that their expressions exhibit a positive correlation in primary tumors. CTGF was also identified as a direct target for YAP1 in other cancer types, such as malignant mesothelioma^21^, hepatocellular carcinoma^22^, and colorectal cancer^23^. Several studies have underscored the proliferation-promoting role of CTGF in cancer. CTGF functions as an oncogene by down-regulating E-cadherin expression via activation of NF-κB pathway^24^, enhancing cyclin D1/MMP-2/MMP-9 expression^25^, and inducing epithelial-to-mesenchymal transition^26^. IHC on tissue microarray from our cohort and TCGA cohort by RNA-seq both identified CTGF as a prognostic marker and its strong expression predicted poor outcome and correlated with advanced stage in GC samples, which was consistent with former reports^25,27^. The upstream members of Hippo pathway, MST1/2, and LATS1/2, negatively regulate YAP1 expression. These tumor suppressor proteins phosphorylate YAP1 on S127 and inhibit YAP1 translocation to the nucleus, thus quenching its transcription activity on CTGF^28,29^. However, due to the epigenetic modification of Hippo pathway, YAP1/TEADs-CTGF cascade is frequently activated and promotes tumorigenesis in many cancers ^30,31^, including GC ^20^.

Apart from Hippo pathway, miRNAs also play an important synergistic role in the regulation of YAP1/TEADs-CTGF cascade. miRNAs have been validated as crucial players in gastric carcinogenesis through post transcriptional regulation of tumor suppressor genes and oncogenes^32^. In our previous study, we confirmed that YAP1 is negatively regulated by miR-15/16 family^9^. In this study, we provided the first evidence that miR-375 is a super-controller for YAP1/TEADs-CTGF cascade by directly targeting YAP1, TEAD4, and CTGF. Although targeting of YAP1 by miR-375 has been reported in liver cancer^33^, lung cancer^34^, and colorectal cancer^35^, our findings revealed miR-375 not only targets YAP1, but also targets its binding partner TEAD4 and its downstream effector CTGF, suggesting a substantial role of miR-375 in the regulation of the Hippo pathway.

In our study, miR-375 was discovered to be expressed in normal gastric epithelium, but was consistently down-regulated across a panel of GC cell lines due to epigenetic silencing, suggesting a potential role in cell transformation. Ectopic expression of miR-375 exerted a tumor-suppressive function by inhibiting GC cell proliferation both in vitro and in vivo. This result was concordant with previous reports of miR-375 in GC^13,15^. Noteworthily, the stomach tumors possess heterogeneity and the protein regulation system is quite complicated. For example, CTGF is not only regulated by YAP1-TEAD complex which directly binds on its promoter region, it is also a target of miR-375 and miR-124. This might be the reason for the inconsistence of the absolute YAP-CTGF positive correlation in primary samples. According to Fig. 2e, we defined the 6th, 7th, 8th, 10th, 12th, 13th, 14th, 15th, 18th, 21st, 22nd, 23rd, and 24th samples as strong-correlation group, and the rest as weak-correlation group based on ImageJ density quantification. We found miR-375 expression is significantly lower in strong-correlation group. Thus, we concluded that miR-375 expression is specifically lower in tumors with strong YAP-CTGF positive correlation.

Apart from GC, miR-375 has been reported as a well-known tumor-suppressive miRNA in human cancers such as breast cancer^36^, esophageal squamous cell carcinoma^37^, colorectal cancer^38^, hepatocellular carcinoma^39^, and pancreatic carcinoma^40^. PDK1^40,41^, JAK2^13^, PIK3CA^38^, SHOX2^36^, IGF1R^37^, and AEG-1^39^ are the main targets of miR-375. In the current study, we unraveled novel targets of miR-375 including YAP1, TEAD4, and CTGF, enriching the target pool of miR-375 in carcinogenesis.

Collectively, YAP1/TEADs-CTGF cascade is activated and promotes progression of GC partly due to the epigenetic silence of the upstream Hippo pathway. miRNAs with tumor-suppressive function, especially miR-375, also play a crucial role in the activation of YAP1/TEADs-CTGF cascade. miR-375 is down-regulated in GC because of its promoter methylation^42^, which lead to its losing inhibitory effect on YAP1, TEAD4, and CTGF. Thus, YAP1/TEADs-CTGF is co-activated to promote gastric carcinogenesis. Our findings not only enhance our understanding of the Hippo pathway and deregulated miRNA network in GC development, but also lead to the identification of several useful biomarkers for predicting GC prognosis.

## Materials and methods

### GC cell lines and primary gastric tissues

Human GC cell lines (MKN1, MKN7, MKN28, MKN45, SNU1, SNU16, AGS, Kato III, NCI-N87, MGC-803, SGC-7901) were maintained in RPMI-1640 medium (Gibco, Grand Island, NY) supplemented with 10% fetal bovine serum (Gibco) in a humidified atmosphere containing 5% carbon dioxide at 37 °C, as previously reported^8^. A tissue microarray was constructed from 145 primary GC samples retrieved from the tissue bank of Anatomical and Cellular Pathology, Prince of Wales Hospital, The Chinese University of Hong Kong. A total of 76 paired RNA samples were extracted from frozen tissues. The use of human samples was approved by Joint Chinese University of Hong Kong–New Territories East Cluster Clinical Research Ethics Committee, Hong Kong. The normal tissue representing to human stomach total RNA was commercially available from Ambion (AM7996, Grand Island, NY). TCGA dataset was retrieved from its official website: http://cancergenome.nih.gov/.

### Treatment of cell lines with 5-Aza, TSA, and VP

AGS, NCI-N87, MGC-803, and MKN1, in which miR-375 was down-regulated, were treated with demethylating agent (5-Aza) and histone deacetylases inhibitor^43^. For 5-Aza (Sigma, St Louis, MO) treatment group, the cells were treated with 10 μM 5-Aza for 3 days. For TSA (Sigma) treatment group, 100 nM TSA was added to the cells for 24 h. For the combination treatment, the cells were treated with 5-Aza for 4 days and in the last 24 h, 100 nM TSA was added. The control cultures were treated with an equal amount of vehicle DMSO (Sigma).

Verteporfin (also named VP, Selleckchem, Houston, TX), a small molecule inhibitor of YAP1-TEAD association which inhibits YAP1’s oncogenic property was used in MKN28, AGS, MGC-803, and SGC-7901 cells to investigate the effect of pharmacological inhibition of YAP1^44,45^. The cells were treated with VP in 0, 1, 2, 5, 10 μM concentrations for a 3-day MTT assay. For the Western blot analysis of YAP1 and CTGF, the protein was collected in 0, 1, 2 μM of VP treatment for 24 h.

### RNA extraction and quantitative real-time polymerase chain reaction (qRT-PCR)

Total RNA from fresh tissue samples and cultured cells was extracted using TRIzol reagent (Invitrogen, Carlsbad, CA). High-Capacity cDNA Reverse Transcription Kits (Applied Biosystems, Carlsbad, CA) were employed for cDNA synthesis. qRT-PCR was used to quantify mRNA levels and primers were listed in Supplementary Table S5. The relative expression level was normalized with B2M (β-2-microglobulin) and calculated using the 2^ (-Delta Delta Ct) method. PCR was performed using SYBR Green PCR reagents (Applied Biosystems) according to the manufacturer’s instructions. The reactions were incubated in a 96-well plate at 95 °C for 10 min, followed by 40 cycles of 95 °C for 15 s and 60 °C for 1 min.

For miRNA expression detection, Taqman miRNA assays were used to quantify the expression of mature miR-375 (KIT, 000564, Applied Biosystems). The relative expression level of microRNAs was normalized by RNU6B (KIT, 001093, Applied Biosystems). The reactions were performed in 7500 Fast Real-Time System (Applied Biosystems) and the reaction mix was incubated at 95 °C for 30 s, followed by 40 cycles of 95 °C for 8 s and 60 °C for 30 s^11^.

### Protein extraction, Western blot analysis and co-immunoprecipitation assays

YAP1 was detected with a monoclonal anti-YAP1 antibody (1:10000 dilution, ab52771, Abcam, Cambridge, MA). CTGF(L-20) antibody (1:1000, sc-14939, Santa Cruz, Dallas, TX), TEAD1 (1:1000, sc-376113) and TEAD4 (1:1000, sc-101184) were also provided by Santa Cruz. Other primary antibodies are from Cell Signaling (Danvers, MA) commercially including p-Rb(Ser807/811) (1:1000, #9308), p21 (1:1000, #2946), p27 (1:1000, #2552), cleaved PARP(Asp214) (1:1000, #9541), p-p44/42 MAPK (1:1000, #9106), p-AKT(S473) (1:1000, #9271), c-Myc (1:1000, #9402), CDK4 (1:1000, #12790), and CDK6 (1:1000, #3136). The other antibodies are active-β-catenin (1:1000, #05-665, Millipore, Billerica, MA) and β-catenin (1:10000, #610154, BD Transduction Laboratories, San Jose, CA). FLAG antibody (YM3001) was available from ImmunoWay GAPDH expression was used as equal loading control. The secondary antibodies are anti-Mouse IgG-HRP (1:30000, 00049039, Dako, Glostrup, Denmark) and anti-Rabbit IgG-HRP (1:10000, 00028856, Dako). The Western blot bands were quantified by ImageJ.

For the co-immunoprecipitation, AGS cells were either transfected with Empty vector or Flag-YAP1 and were lysed 48 h later in lysis buffer (50 mM Tris-HCl pH 7.4, 150 mM NaCl, 1% Triton) containing protease inhibitor cocktails (Roche, Indiana, USA). 500 µg total protein was used for immunoprecipitation with 20 µl ANTI-FLAG M2 affinity gel (A2220, Sigma). After incubation at 4 °C for 4 h, immunoprecipitates were washed 3 times and resuspended in 20 µl SDS loading buffer, then resolved by SDS-PAGE after heating at 98 °C for 5 min. As the signals generated is close to the heavy chain (~50 kDa), light chain specific secondary antibody from Abcam (ab99632) was used for Western blot.

### Immunohistochemistry (IHC) and scoring

For IHC, the primary antibodies (1:50 for CTGF and 1:1000 for YAP1) were incubated at 4 °C overnight and chromogen development was performed using the EnVision system (Dako). The cytoplasmic expression of CTGF was assessed by assigning a labeling index, which was a proportion score multipled by an intensity score. The proportion score referred to proportion of tumor cells with positive cytoplasmic staining (0, none; 1, <10%; 2, 10–≤25%; 3, >25–50%; 4, >50%), whereas the intensity score represented the average intensity of positive tumor cells (0, none; 1, weak; 2, intermediate; 3, strong). The labeling index of CTGF was categorized into negative, score 0; weak, score 1, 2, 3; moderate, score 4, 6; and strong, score 8, 9, 12. Nuclear YAP1 scoring was detailed described in previous report^8^.

### miRNA, anti-miRNA, siRNA, plasmid transfection, and in vitro functional study

miR-375 precursor (AM17100, ID: PM10327, Life Technologies), scramble control (AM17110), Anti-miR-375 (AM17000, ID: AM10327), and Anti-miRNA control (AM17010) were purchased from Thermo Fisher Scientific. As for siRNA-meditated gene knockdown, siCTGF (SI00029673), siYAP1 (SI02662954), siTEAD1 (SI04181261), siTEAD4 (SI04131127), and AllStars Negative Control siRNA (SI03650318) were obtained from Qiagen (Valencia, CA). The transfection concentration is 25 nM. The functional studies are as follows.

Cell proliferation was assessed using CellTiter 96 Non-Radioactive Cell Proliferation Assay (Promega, Madison, WI) according to manufacturer’s instruction. For colony formation assays in monolayer cultures, transfected cells were cultured for 10 days, fixed with 70% ethanol for 15 min and stained with 2% crystal violet. Colonies with more than 50 cells per colony were counted. The cell invasion assays were conducted using Biocoat Matrigel Invasion Chambers (BD Biosciences, Franklin Lakes, NJ) as described previously^8^. The cell migration assays were performed by Transwell Polycarbonate Membrane Inserts (Corning, NY). Cells that invaded through the gel and adhered to the bottom side of the chambers were counted. All the cell functional experiments were performed three times independently to get standard deviations (SDs). The cell cycle analysis had been described in detail in the previous report^9^. In the senescence experiments, AGS, NCI-N87, and MGC-803 cells were transfected with miR-375 or negative control for 3 days at 25 nM concentration. Then the cells were stained with β-Galactosidase (Kit, #9860, Cell Signaling) for 8 h and the positive cell population showed pale green under the microscope. The positive cell was counted and the standard deviation was achieved by calculating the ratio of positive cells per 100 cancer cells in 3 random vision fields. In the rescue experiments, YAP1 was subcloned into Empty vector (pcDNA3, Life Technologies) and transfection was conducted by FuGENE HD Transfection Reagent (Roche, Nutley, NJ).

### ChIP-PCR and ChIP-qPCR

Before ChIP-PCR, 14 putative targets genes (BCCIP, CCDC80, CTNNB1, MBNL2, MTUS1, SCO1, TMEM165, MMP-26, CTGF, AXL, INHBA, TRPC1, JAK2, and SMO) were screened out from our in-house ChIP-Seq data (genes with the most significant *P*-value) and other reported database^46^. We found CTGF was the only gene showing down-regulated expression in all four GC cell lines examined after YAP1 knockdown. For ChIP-PCR and qPCR analysis, the primers targeting a region within 200 bp of the putative binding site in CTGF promoter were designed (sense: TTC TGT GAG CTG GAG TGT GC; antisense: GCC AAT GAG CTG AAT GGA GT). An equal amount of DNA samples from IP (by YAP1 antibody or IgG antibody as a negative control) was used as a template for conventional PCR assay.

### Luciferase assays

The putative miR-375 binding sites in 3’UTR of YAP1 (two binding sites), TEAD4, and CTGF were subcloned into a pMIR-REPORT vector (Ambion). Meanwhile, the corresponding mutant constructs were generated by mutation of the complementary sequence of miR-375 seed region. The sense and antisense of oligonucleotides (Supplementary Table S6) that encompassed the miR-375 binding sites were annealed and subcloned into pMIR-REPORT vector^47^. Bioinformatics analysis revealed CTGF promoter region encompasses three consecutive TEAD (binding partner of YAP1) binding motifs. Thus, a YAP1-bound promoter region of CTGF (200, 600, 1000 bp from the transcription starting site of CTGF respectively) was subcloned into the reporter gene vector pGL3-Basic (Promega). The firefly luciferase construct was co-transfected with Renilla luciferase vector (Promega) control into the cells. Dual Luciferase Reporter Assay System (Promega) was employed to check the luciferase activity after 1-day transfection.

### In vivo tumorigenicity study

MGC-803 cells (10^7^ cells suspended in 0.1 ml PBS) transiently transfected with Negative control (empty vector) or miR-375 stable-expression plasmid were injected subcutaneously into the left and right dorsal flank of 4-week old Balb/c nude mice respectively. Tumor weights (g) were calculated 25 days after inoculation. The rescue experiments and CTGF knockdown assay by animal model were the same as miR-375 in vivo study. All animal handling and experimental procedures were approved by Department of Health, Hong Kong (Reference No: 14-267 in DH/HA&P/8/2/1 Pt.38) and Animal Experimentation Ethics Committee, The Chinese University of Hong Kong.

### Statistical analysis

The paired *t*-test was used to compare the difference in biological behavior between miR-375 transfected cells and scramble miRNA control transfected cells. It was also employed to compare CTGF knockdown cells and scramble siRNA-transfected counterparts. Expression of miR-375 in primary cancerous tissues and the corresponding paired noncancerous tissues were compared by paired T test. Correlation between miR-375 or CTGF expression and clinicopathologic parameters were assessed by nonparametric Pearson Chi-Square test. The Kaplan–Meier method was used to estimate the survival rate for each parameter. The equivalences of the survival curves were tested by log-rank statistics. For those variables were found statistically significant in the univariate survival analysis (*P* *<* 0.05), the Cox proportional hazards model with the likelihood ratio statistics was employed to further evaluate them for multivariate survival analysis. All statistical analysis was performed by SPSS software (Version 22.0; SPSS Inc). A two-tailed *P-*value of less than 0.05 was considered statistically significant and the *P*-value less than 0.001 was considered highly significant.

## Electronic supplementary material

Supplementary Table S1

Supplementary Table S2

Supplementary Table S3

Supplementary Table S4

Supplementary Table S5

Supplementary Table S6

Supplementary Figure S1

Supplementary Figure S2

Supplementary Figure S3

Supplementary Figure S4
