# Supplementary material for: miR-375 is involved in Hippo pathway by targeting YAP1/TEAD4-CTGF axis in gastric carcinogenesis
Source: Cell Death Dis. 2018 Jan 24;9(2):92. doi: 10.1038/s41419-017-0134-0 (PMC5833783; doi:10.1038/s41419-017-0134-0)
Supplement: Supplementary file 1 — Supplementary Table S1 [file 41419_2017_134_MOESM1_ESM.doc]

**Table S1.** Correlation of miR-375 expression with other clinicopathologic parameters in GC (n = 76, significant *P*-value in bold and Italic format).

|  |  | miR-375 expression (/RNU6B, log2) | | |
| --- | --- | --- | --- | --- |
|  |  | >-2, high expression, (%) | <-2, low expression, (%) | *P*-value |
| Sex | F | 9 (25.7) | 26 (74.3) | 0.149 |
|  | M | 18 (43.9) | 23 (56.1) |  |
| Age | <=60 | 10 (41.7) | 14 (58.3) | 0.454 |
|  | >60 | 17 (32.7) | 35 (67.3) |  |
| Type | Intestinal | 13 (40.6) | 19 (59.4) | 0.473 |
|  | Diffuse | 14 (31.8) | 30 (68.2) |  |
| Grade | 1 | 0 (0.0) | 1 (100.0) | 0.736 |
|  | 2 | 18 (35.3) | 33 (64.7) |  |
|  | 3 | 8 (38.1) | 13 (61.9) |  |
| Stage | 1 | 14 (70.0) | 6 (30.0) | ***0.002*** |
|  | 2 | 7 (30.4) | 16 (69.6) |  |
|  | 3 | 4 (22.2) | 14 (77.8) |  |
|  | 4 | 2 (13.3) | 13 (86.7) |  |
| Lymph node | 0 | 14 (66.7) | 7 (33.3) | ***0.001*** |
|  | 1 | 13 (23.6) | 42 (76.4) |  |
| *H. pylori* | Absence | 20 (35.1) | 37 (64.9) | 0.759 |
|  | Presence | 4 (26.7) | 11 (73.3) |  |
