# Supplementary material for: miR-375 is involved in Hippo pathway by targeting YAP1/TEAD4-CTGF axis in gastric carcinogenesis
Source: Cell Death Dis. 2018 Jan 24;9(2):92. doi: 10.1038/s41419-017-0134-0 (PMC5833783; doi:10.1038/s41419-017-0134-0)
Supplement: Supplementary file 2 — Supplementary Table S2 [file 41419_2017_134_MOESM2_ESM.doc]

**Table S2.** Univariate and multivariate Cox regression analysis of clinicopathologic factors with disease specific survival in GC patients (n = 76, significant *P*-value in bold and Italic format; CI: confident interval).

|  | Univariate | Hazard Ratio (95% CI) | Multivariate | Hazard Ratio (95% CI) |
| --- | --- | --- | --- | --- |
| Sex | ***0.030*** | 0.493 (0.260~0.935) | 0.738 | 1.13 (0.550~2.327) |
| Age | 0.143 | 1.719 (0.833~3.549) |  |  |
| Type | ***0.005*** | 2.781 (1.353~5.716) | ***0.041*** | 2.327 (1.036~5.226) |
| Grade | 0.887 | 1.052 (0.525~2.107) |  |  |
| Stage | ***< 0.001*** | 2.619 (1.868~3.672) | ***<0.001*** | 2.221 (1.437~3.432) |
| Lymph node | ***0.002*** | 5.094 (1.796~14.444) | 0.672 | 1.325 (0.360~4.879) |
| *H. pylori* | 0.189 | 1.632 (0.786~3.388) |  |  |
| miR-375 | ***0.004*** | 0.397 (0.210~0.751) | ***0.023*** | 0.443 (0.220~0.890) |
