# Supplementary material for: miR-375 is involved in Hippo pathway by targeting YAP1/TEAD4-CTGF axis in gastric carcinogenesis
Source: Cell Death Dis. 2018 Jan 24;9(2):92. doi: 10.1038/s41419-017-0134-0 (PMC5833783; doi:10.1038/s41419-017-0134-0)
Supplement: Supplementary file 3 — Supplementary Table S3 [file 41419_2017_134_MOESM3_ESM.doc]

**Table S3.** Correlation of CTGF cytoplasmic expression in GC with other clinicopathologic features (significant *P*-value in bold and Italic format).

|  |  | Gastric cancer (n = 145) | | |
| --- | --- | --- | --- | --- |
|  |  | negative/weak number (%) | moderate/strong number (%) | *P*-value |
| Sex | M | 16 (17.6) | 75 (82.4) | 0.889 |
|  | F | 9 (16.7) | 45 (83.3) |  |
| Age | <=60 | 10 (22.7) | 34 (77.3) | 0.251 |
|  | >60 | 15 (14.9) | 86 (85.1) |  |
| Type | Intestinal | 15 (17.2) | 72 (82.8) | 1.000 |
|  | Diffuse | 10 (17.2) | 48 (82.8) |  |
| Grade | 1 | 2 (28.6) | 5 (71.4) | 0.601 |
|  | 2 | 7 (12.1) | 51 (87.9) |  |
|  | 3 | 16 (20.0) | 64 (80.0) |  |
| Stage | 1 | 5 (26.3) | 14 (73.7) | 0.067 |
|  | 2 | 6 (31.6) | 13 (68.4) |  |
|  | 3 | 7 (13.2) | 46 (86.8) |  |
|  | 4 | 7 (13.0) | 47 (87.0) |  |
| Stage (Early/Advanced) | Early(1&2) | 11 (28.9) | 27 (71.1) | ***0.026*** |
|  | Advanced(3&4) | 14 (13.1) | 93 (86.9) |  |
| Stage (T) | 1 | 2 (22.2) | 7 (77.8) | 0.185 |
|  | 2 | 9 (25.0) | 27 (75.0) |  |
|  | 3 | 13 (14.1) | 79 (85.9) |  |
|  | 4 | 1 (12.5) | 7 (87.5) |  |
| Stage (N) | 0 | 6 (28.6) | 15 (71.4) | 0.067 |
|  | 1 | 9 (23.1) | 30 (76.9) |  |
|  | 2 | 5 (10.4) | 43 (89.6) |  |
|  | 3 | 5 (13.5) | 32 (86.5) |  |
| Stage (M) | 0 | 22 (18.0) | 100 (82.0) | 0.564 |
|  | 1 | 3 (13.0) | 20 (87.0) |  |
| Lymph Node | 0 | 6 (28.6) | 15 (71.4) | 0.139 |
|  | 1 | 19 (15.3) | 105 (84.7) |  |
| *H. pylori* | Absence | 8 (16.0) | 42 (84.0) | 0.637 |
|  | Presence | 16 (19.3) | 67 (80.7) |  |
