# Supplementary material for: miR-375 is involved in Hippo pathway by targeting YAP1/TEAD4-CTGF axis in gastric carcinogenesis
Source: Cell Death Dis. 2018 Jan 24;9(2):92. doi: 10.1038/s41419-017-0134-0 (PMC5833783; doi:10.1038/s41419-017-0134-0)
Supplement: Supplementary file 4 — Supplementary Table S4 [file 41419_2017_134_MOESM4_ESM.doc]

**Table S4.** Univariate and multivariate Cox regression analysis of the association between clinicopathologic characteristics and disease specific survival in patients with gastric adenocarcinoma (n = 145, significant *P*-value in bold and Italic format; CI: confident interval).

|  | Univariate analysis | Hazard Ratio (95% CI) | Multivariate analysis | Hazard Ratio (95% CI) |
| --- | --- | --- | --- | --- |
| Sex | ***0.004*** | 1.859 (1.217~2.839) | ***0.005*** | 1.904 (1.219~2.973) |
| Age | 0.992 | 1.002 (0.642~1.564) |  |  |
| Type | ***0.002*** | 1.937 (1.272~2.949) | 0.498 | 0.831 (0.488~1.418) |
| Grade | ***0.007*** | 1.668 (1.149~2.421) | 0.418 | 1.224 (0.751~1.995) |
| Stage | ***<0.001*** | 2.654 (2.011~3.504) | ***<0.001*** | 2.617 (1.808~3.788) |
| Stage (T) | ***<0.001*** | 2.644 (1.839~3.802) |  |  |
| Stage (N) | ***<0.001*** | 1.977 (1.576~2.479) |  |  |
| Stage (M) | ***<0.001*** | 5.279 (3.063~9.100) |  |  |
| Lymph Node | ***<0.001*** | 5.532 (2.231~13.718) | 0.848 | 0.892 (0.277~2.870) |
| *H. pylori* | 0.150 | 0.717 (0.456~1.127) |  |  |
| CTGF | ***0.027*** | 2.109 (1.090~4.083) | 0.188 | 1.573 (0.802~3.084) |
