# Supplementary material for: miR-375 is involved in Hippo pathway by targeting YAP1/TEAD4-CTGF axis in gastric carcinogenesis
Source: Cell Death Dis. 2018 Jan 24;9(2):92. doi: 10.1038/s41419-017-0134-0 (PMC5833783; doi:10.1038/s41419-017-0134-0)
Supplement: Supplementary file 5 — Supplementary Table S5 [file 41419_2017_134_MOESM5_ESM.doc]

**Table S5. Primers used in this study.**

| Primer | Sense (5’-3’) | Antisense (5’-3’) |
| --- | --- | --- |
| YAP1 | CAGCAACTGCAGATGGAGAA | ACATCCCGGGAGAAGACACT |
| TEAD4 | TCCACGAAGGTCTGCTCTTT | GTGCTTGAGCTTGTGGATGA |
| CTGF | GGGCCTATTCTGTCACTTCG | ACGTGCACTGGTACTTGCAG |
| B2M | ACTCTCTCTTTCTGGCCTGG | ATGTCGGATGGATGAAACCC |
