# Supplementary material for: miR-375 is involved in Hippo pathway by targeting YAP1/TEAD4-CTGF axis in gastric carcinogenesis
Source: Cell Death Dis. 2018 Jan 24;9(2):92. doi: 10.1038/s41419-017-0134-0 (PMC5833783; doi:10.1038/s41419-017-0134-0)
Supplement: Supplementary file 6 — Supplementary Table S6 [file 41419_2017_134_MOESM6_ESM.doc]

**Table S6.** Oligonucleotides used in the luciferase activity experiments. The oligonucleotides were annealed and subcloned into pMIR-REPORT via *HindIII* and *SpeI* restriction sites. Wild type, full length of the putative miR-375 binding site; Mutation, the binding site was mutated; BS1, binding site 1; BS2, binding site 2.

|  | Oligonucleotides |  |
| --- | --- | --- |
| YAP1 | Wild type (BS1) Sense | 5’ CT AGT AAT ACA GAA AAA GAT GAA CAA ACA 3’ |
|  | Wild type (BS1) Antisense | 5’ AG CTT GTT TGT TCA TCT TTT TCT GTA TTA 3’ |
|  | Mutation (BS1) Sense | 5’ CT AGT AAT ACA GAA AAA GAT CTT GTT TCA 3’ |
|  | Mutation (BS1) Antisense | 5’ AG CTT GAA ACA AGA TCT TTT TCT GTA TTA 3’ |
|  | Wild type (BS2) Sense | 5’ CT AGT GAG TAT TTT TTA AAG GAA CAA AAA 3’ |
|  | Wild type (BS2) Antisense | 5’ AG CTT TTT TGT TCC TTT AAA AAA TAC TCA 3’ |
|  | Mutation (BS2) Sense | 5’ CT AGT GAG TAT TTT TTA AAG CTT GTT TAA 3’ |
|  | Mutation (BS2) Antisense | 5’ AG CTT TAA ACA AGC TTT AAA AAA TAC TCA 3’ |
| TEAD4 | Wild type Sense | 5’ CT AGT TGA CTC TAC CCA GGA ACA AAC TA 3’ |
|  | Wild type Antisense | 5’ AG CTT AGT TTG TTC CTG GGT AGA GTC AA 3’ |
|  | Mutation Sense | 5’ CT AGT TGA CTC TAC CCA GCT TGT TTC TA 3’ |
|  | Mutation Antisense | 5’ AG CTT AGA AAC AAG CTG GGT AGA GTC AA 3’ |
| CTGF | Wild type Sense | 5’ CT AGT AGC CTC ACT TTT AAT GAA CAA ATG A 3’ |
|  | Wild type Antisense | 5’ AG CTT CAT TTG TTC ATT AAA AGT GAG GCT A 3’ |
|  | Mutation Sense | 5’ CT AGT AGC CTC ACT TTT AAT CTT GTT TTG A 3’ |
|  | Mutation Antisense | 5’ AG CTT CAA AAC AAG ATT AAA AGT GAG GCT A 3’ |
