# Supplementary figures and images for: miR-375 is involved in Hippo pathway by targeting YAP1/TEAD4-CTGF axis in gastric carcinogenesis
Source: Cell Death Dis. 2018 Jan 24;9(2):92. doi: 10.1038/s41419-017-0134-0 (PMC5833783; doi:10.1038/s41419-017-0134-0)

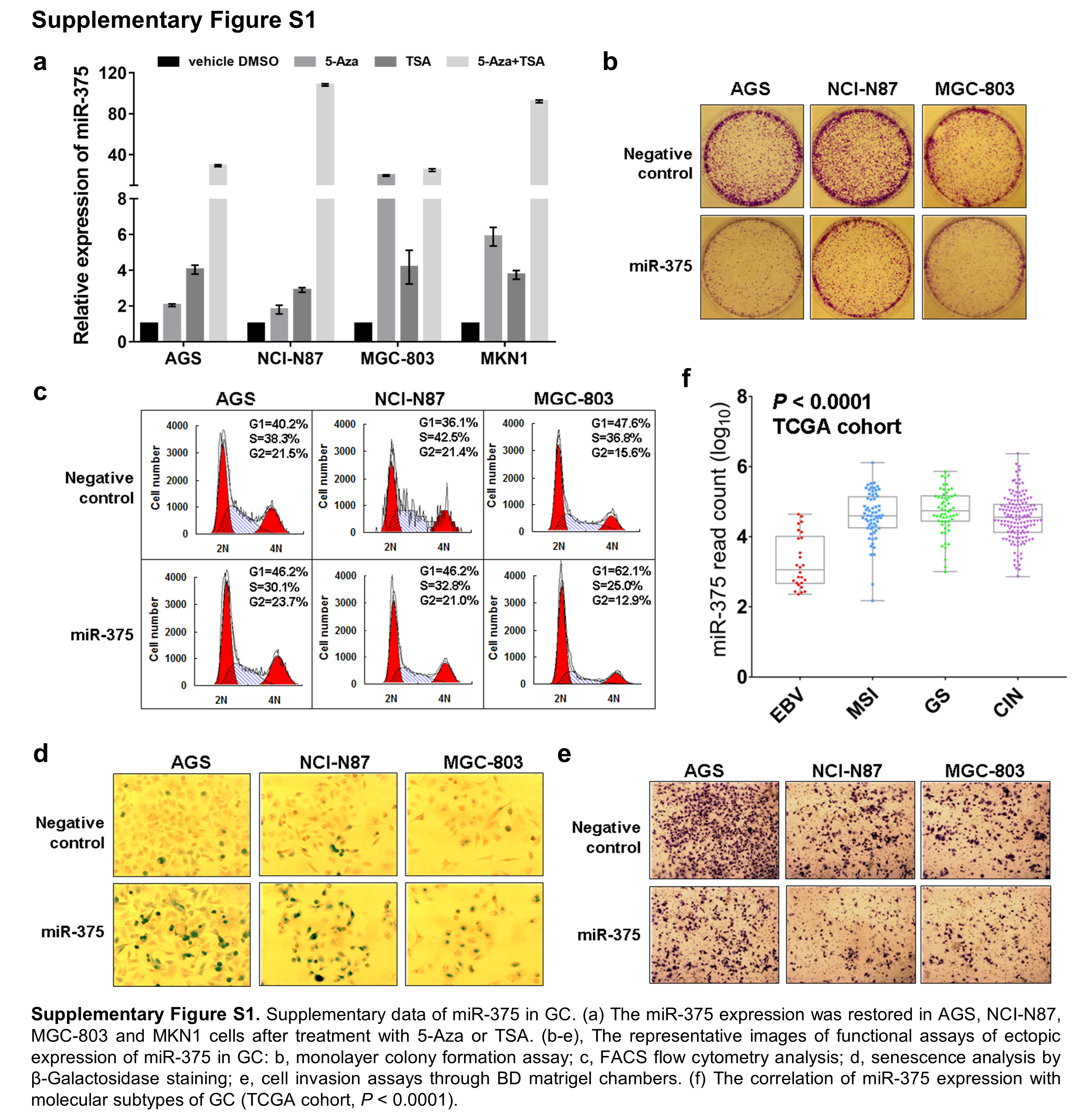

Supplement: Supplementary file 7 — Supplementary Figure S1 [file 41419_2017_134_MOESM7_ESM.tif]

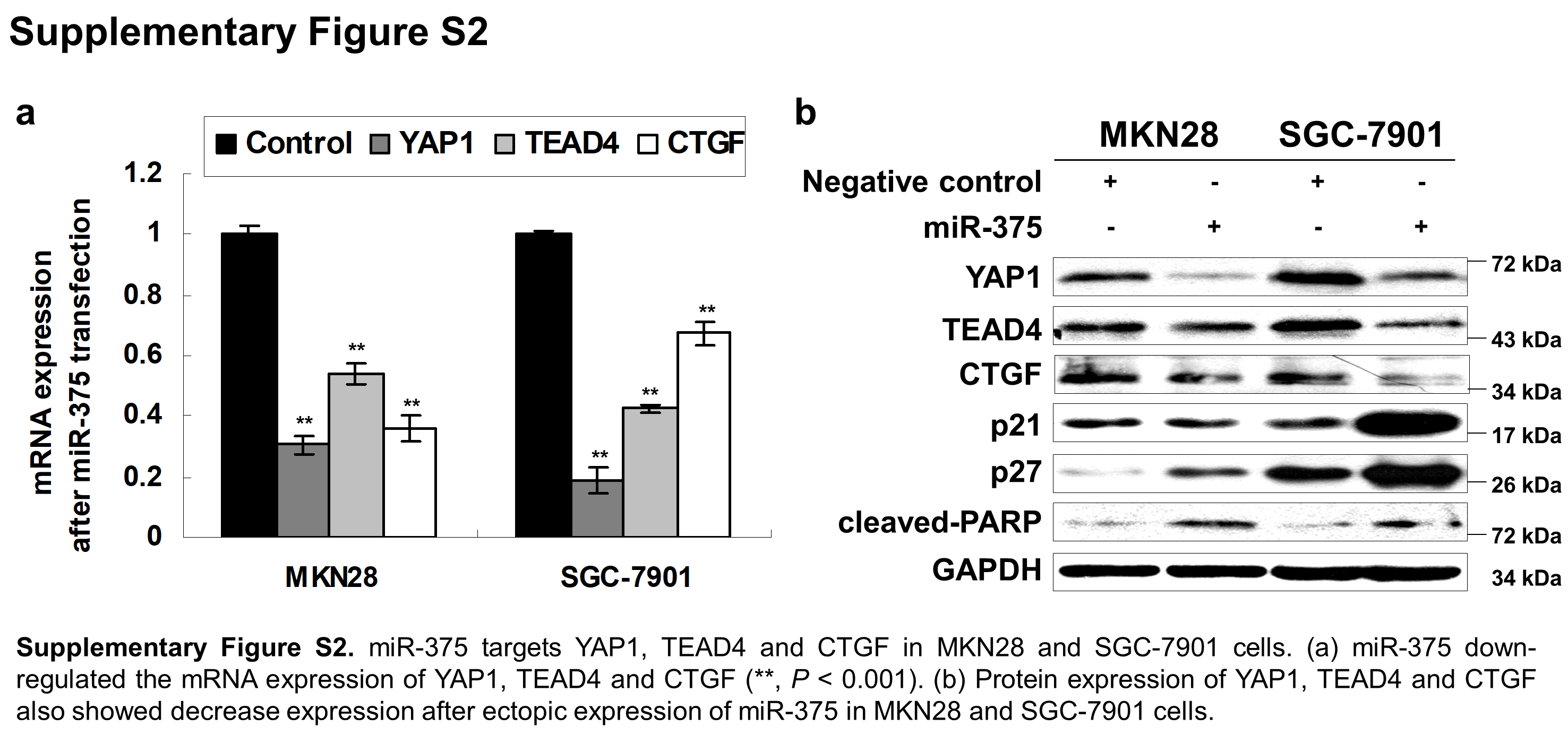

Supplement: Supplementary file 8 — Supplementary Figure S2 [file 41419_2017_134_MOESM8_ESM.tif]

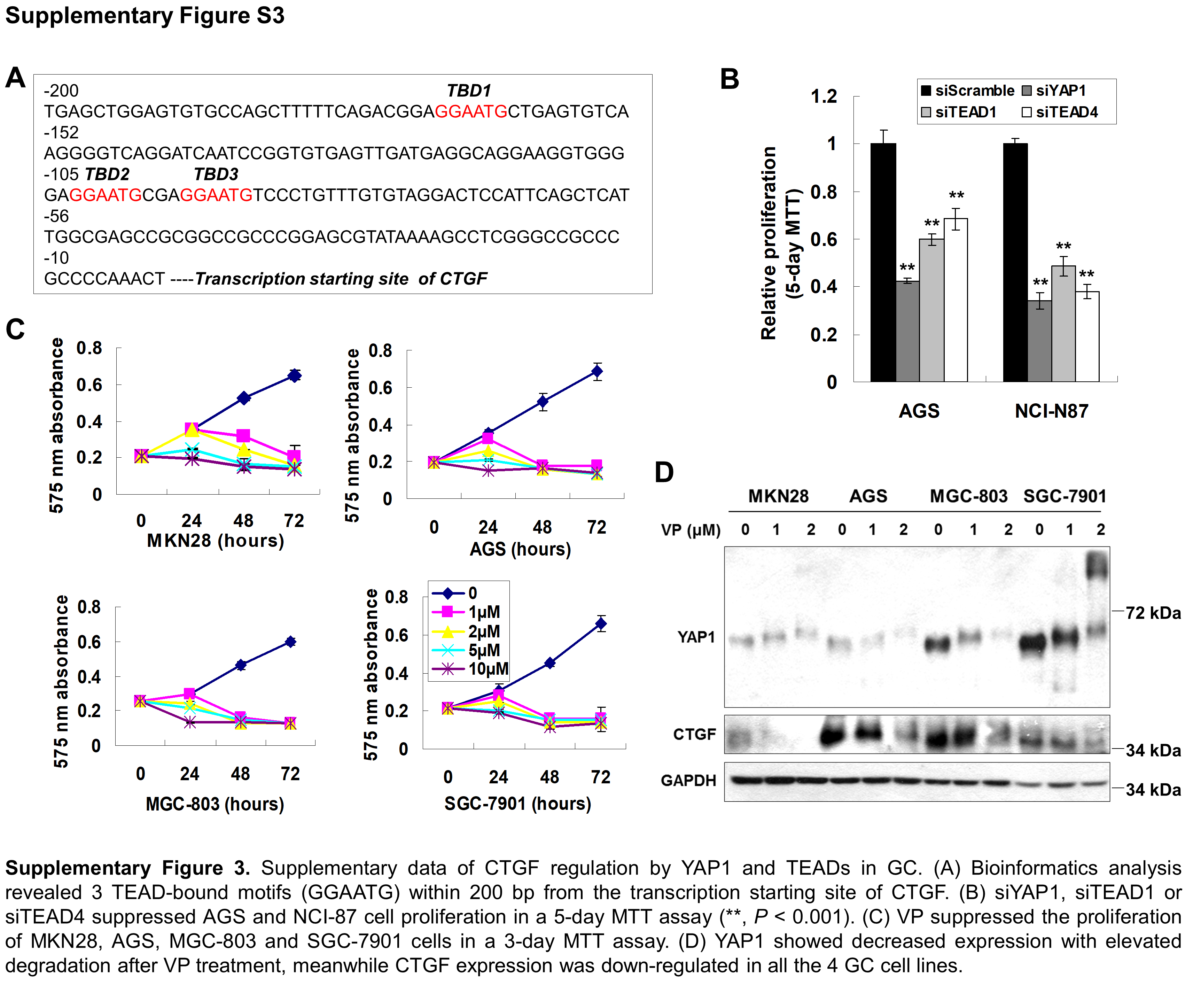

Supplement: Supplementary file 9 — Supplementary Figure S3 [file 41419_2017_134_MOESM9_ESM.tif]

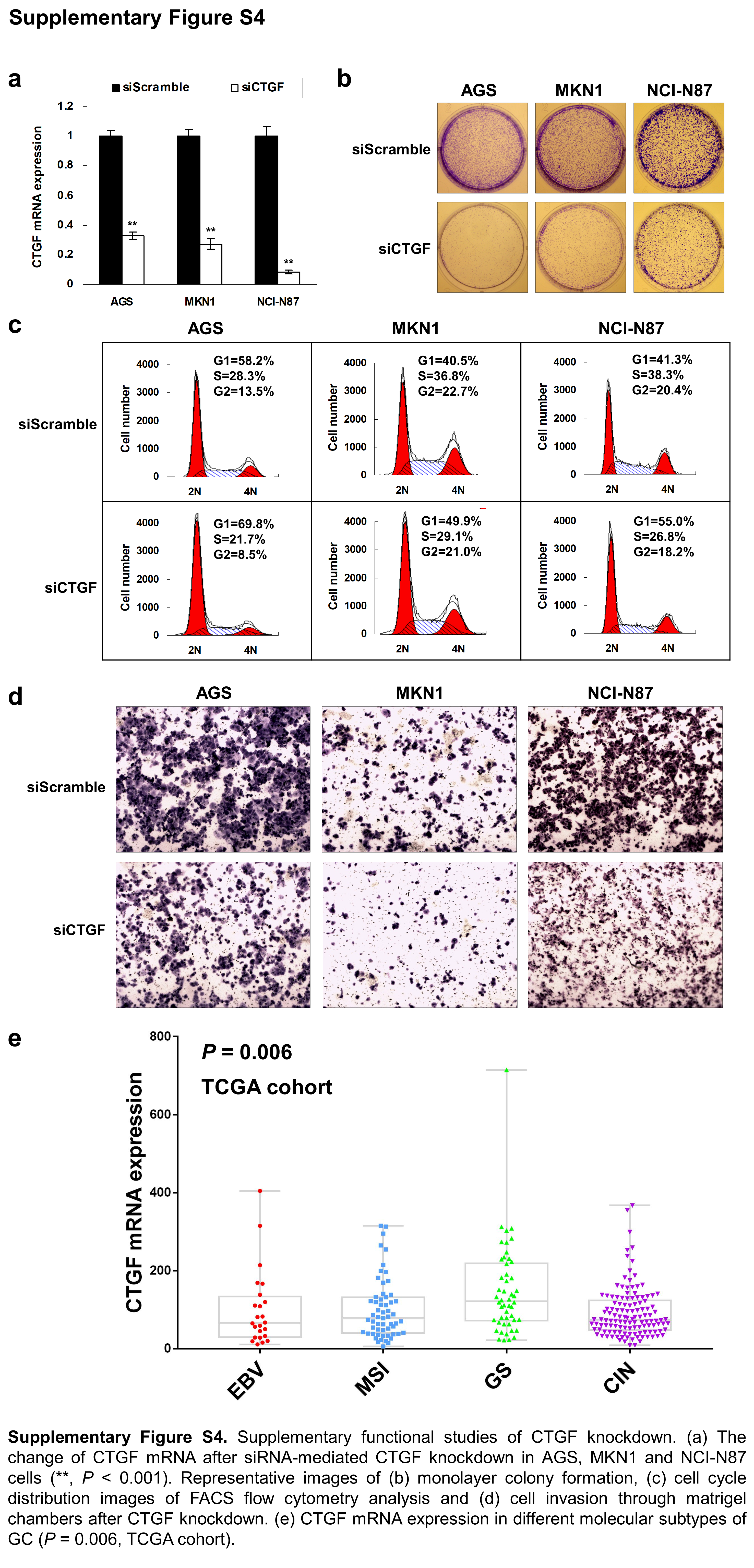

Supplement: Supplementary file 10 — Supplementary Figure S4 [file 41419_2017_134_MOESM10_ESM.tif]
